# The impact of various selenium forms during the maturation period of Gilthead seabream (*Sparus aurata*) broodstock on the reproductive performance, egg, and offspring quality

**DOI:** 10.1038/s41598-025-24555-x

**Published:** 2025-11-18

**Authors:** Alaa A. El-Dahhar, Ahmad Abde-Salam, Samy Y. EL-Zaeem, Mohammed M Abdel-Rahim, Mona M. Mourad

**Affiliations:** 1 Animal and Fish Production Department, Faculty of Agriculture (Saba Bash), Alexandria University, Alexandria, Egypt; 2 General Authority for Fish Resources Development (GAFRD), Marine Fish Hatchery, Cairo, Egypt; 3 National Institute of Oceanography and Fisheries (NIOF), Cairo, 11562 Egypt

**Keywords:** Biochemistry, Ecology, Ecology, Physiology, Zoology

## Abstract

This study investigates the effect of dietary Selenium (Se) supplementation on reproductive performance, fecundity, hatchability, egg and oil droplet size, and serum biochemical parameters in Gilthead Sea Bream (G. Sea Bream, *Sparus aurata*) broodstock. The study includes three treatment groups: a basal diet (BD) without selenium supplementation (control group, C), a BD supplemented with selenium nanoparticles (Nano-Se) at 0.3 mg/kg, and a BD supplemented with organic selenium (Org-Se) at 4 mg/kg (2 g of crushed yeast; Sel-Plex 2000 mg Se kg-1, Alltech, Lexington, KY). The glutathione peroxidase (GPx) enzyme activity, the characteristic of the egg and offspring, was examined, as well as the survival and growth of the resulting larvae. Nine 15 cubic meters fiberglass tanks were used in the experiment, filled with seawater of a salinity of 36.9 ± 1.1 parts per thousand (ppt). Each tank was stocked with six G. seabream broodstock, maintaining an equal sex ratio (three males and three females). The females’ average weight was 1000 ± 50 g, while the males averaged 400 ± 50 g. The brooders were fed 5% of their body weight three times daily for 84 days, using an equal mixture of 45% crude protein dry feed, squid, and sardine. The broodstock were injected with 10 µg/kg of a Luteinizing hormone-releasing hormone (LHRH) analog after the 84-day feeding period to induce spawning. The levels of reproductive hormones, including luteinizing hormone (LH), follicle-stimulating hormone (FSH), testosterone (T), and estradiol (E), in brooders (males and females) fed selenium-supplemented diets with Nano-Se and Org-Se increased. In males fed Nano-Se, the increases compared to the control were 74.8%, 84.8%, and 134.8% for the respective groups. While the males fed Org-Se, the increases were 14.01%, 20.01%, and 26.09%, respectively. However, in Females fed the Nano-Se diet, increases as compared to control were 58.1%, 121.4%, and 98.5%, and in females fed the Org-Se diet, increases were 28.17%, 38.21%, and 55.22% for LH, FSH, and estradiol (E), respectively. Albumin, total protein, and globulin levels, as compared to the control, were also increased in brooders (both male and female) fed the Nano and Organic Selenium diets. In contrast, the brooders (male and female) fed the Nano and Organic Selenium diets exhibited triglycerides, lipids, and cholesterol lower than the control. The total egg number, relative fecundity, final egg and oil droplet diameter, and hatchability were also higher in the brooders fed the Nano and Organic Selenium diets than in the control. Data of the larvae (20 DPH) produced from broodstock fed with diets fortified with different forms of Selenium during the maturation period were also investigated. The larvae from mothers fed the Nano-Se exhibited higher final length, final weight, and survival rates than those fed the Control and Org-Se diets.

## Introduction

Selenium (Se) is vital for fish, as it is essential for their development and critical in preserving biological substances such as lipids, DNA, and proteins^1,2^. Selenium is vital for the function of melanoproteins, defends against free radicals, and is especially important for Se-dependent GPx, protecting cells against oxidative damage. Furthermore, Selenium supports growth and regulates the physiological processes of genes^3–5^. Selenium deficiency can lead to several health problems in fish, whereas excessive levels can result in toxicity. Evaluating the type and concentration of Selenium in fish feed is crucial to ensure its bioavailability while minimizing excessive amounts. The EFSA European Authority suggests a maximum Selenium limit for animal feeds and recommends specific Selenium supplementation to support optimal antioxidant levels in fish^6^. Furthermore, new organic Selenium variants, such as hydroxy selenomethionine (OH-Se-Met), have been introduced to enhance Selenium availability.

This enzyme complex, comprising multiple isoenzymes, utilizes glutathione to reduce peroxides and hydroperoxides, converting them into alcohols and water, which are more stable^7^. Consequently, GPx activity in the liver has been proposed as a significant indicator of fish’s selenium (Se) levels^3^. For example, a positive relationship between GPx activity of the liver and Se concentration was found in both the liver and the body of channel catfish, as observed by^8^. Likewise, these findings were observed by^9^ in Atlantic salmon and also in grouper, as reported by^10^. In 2007, similar results were also reported for the Gibel carp and the African catfish^11,12^.

Selenium (Se) is vital for proper skeletal development. Consequently, the dietary levels of Se significantly influence the expression of genes that encode bone morphogenetic protein (BMP) and osteocalcin (OC), both of which are essential proteins for bone cell differentiation and mineralization^13,14^. Furthermore, Se is acknowledged for its involvement in controlling the expression of several genes linked to phospholipid synthesis, redox activities, and immune responses^15–17^. A lack of selenium can result in reduced appetite, hindered growth, increased mortality rates, diminished muscle strength, and oxidative harm to cells and membranes due to lower GPX activity and impaired immune responses in fish^2,10,18^. The increased levels of Selenium (Se) in fish can cause high toxicity, like that of terrestrial domestic animals. Selenium toxicity can be associated with alterations in glutathione peroxidase (GPx) levels in cells^19,20^. Nonetheless, the effects of either an increased level or a lake of Se are influenced by several factors, such as the fish species, the Se oxidation state, and the dietary Se level^20,21^.

Selenium is found in various dietary components and can be added explicitly to the diet in different forms. The dietary Se supplementation form influences its bioavailability^22^. Selenium is present in multiple food sources and can be intentionally incorporated into the diet in various forms. Selenium absorption is affected by the dietary format in which it is consumed^3,24,25,25^. Additionally, an elevation in dietary Selenium in its organic form demonstrated enhanced availability and efficacy in the expression of genes related to oxidative stress in sea bream^26^. Consequently, taking into account the significance levels of (E and C vitamins) as antioxidants and the need to keep the status of healthy antioxidants in fish while preventing the increased amounts, and maintaining a maximum Selenium content of 0.5 mg/kg in animal feeds^27^ and 0.2 mg/kg as a maximum supplement for seleno-methionine (Se-Met)^28^. Sodium selenite (NaSe) is a common inorganic Selenium source used as a dietary supplement in animals and fish^29^. However, hydroxy-selenomethionine (OH-Se-Met), in its organic form, has been developed to enhance Selenium availability. This organic Selenium source has been studied for its effectiveness in poultry^30,32,32^. Consequently, in the current study, the impact of Nano-Se and Org-Se on the reproductive performance, fecundity, hatchability, egg diameter, oil droplet size, and serum biochemical parameters in Gilthead Sea Bream (G. Sea Bream, *Sparus aurata*) broodstock was examined. In addition to growth performance, the antioxidant status and stress resistance of gilthead sea bream larvae were also investigated.

## Materials and methods

### The fish husbandry management, broodstock and rearing conditions

**Fig. 1:**
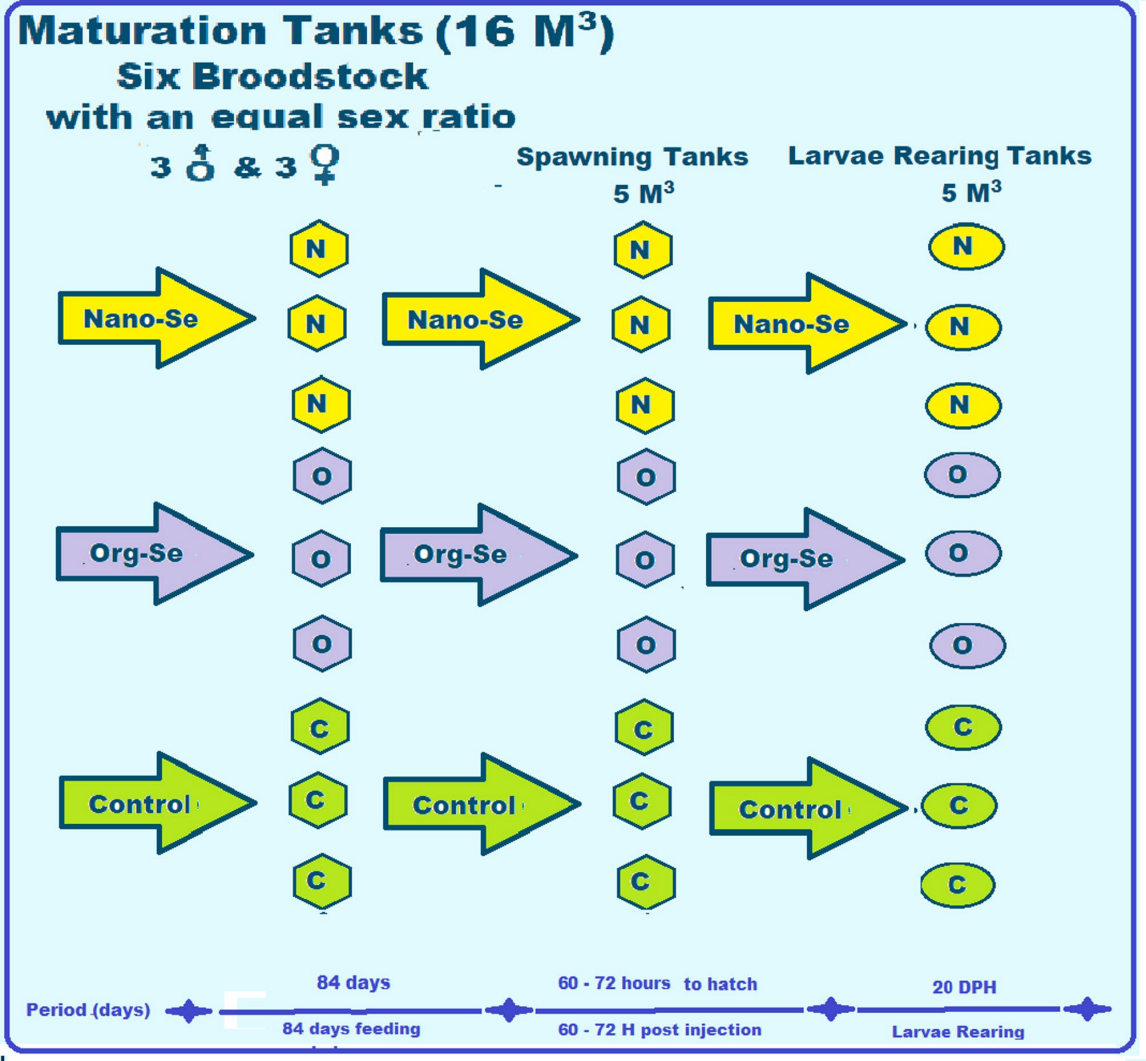
The experimental periods and design (broodstock maturation, spawning, and larvae rearing in days) of Gilthead seabream, *Sparus aurata* broodstock, fed three diets (Nano-Se, Org-Se, and control).

This study involved 54 Gilthead seabream broodstock from the Marine Governmental Hatchery of the General Authority for Fish Resources Development (GAFRD) in Alexandria, Egypt. In the hatchery, the broodstock were housed in nine fiberglass tanks with a total capacity of 20 m^³^ and an active volume of 16 m^³^ of seawater. Each tank contained three females (average weight: 1.0 ± 0.06 kg) and three males (average weight: 0.4 ± 0.10 kg). The seawater supplied to the tanks was filtered through a sand filter, well-aerated, and sourced from the Mediterranean Sea. The experimental periods (broodstock maturation, spawning, and larvae rearing in days) and the design of the present study are shown in Fig. 1.

From October to December, a natural photo period was maintained in the experimental tanks, consisting of 10 h and 15 min of light followed by 13 h and 45 min of darkness. The maturation tanks were located under a greenhouse covered with a black polyethylene sheet. The water exchange rate was set at 200%, equivalent to a flow rate of 22 L per minute.

Throughout the 84-day experimental period, the fish were kept under a natural temperature regime ranging from 16 to 19 °C. The pH ranged from 7.8 to 8.0, and the oxygen content exceeded 5.2 ppm. The un-ionized ammonia level was measured at 0.002 ppm, and salinity levels ranged from 36 to 37 ppt.

### Experimental design and diets

Seabream broodstock were fed a basal diet (BD) composed of an equal mix of dry feed (containing 45% protein), squid, and sardine. This diet was provided at a daily rate of 5%, split into three portions. In the study, three treatments were used: the BD without selenium supplementation (control group, C), the BD with 4 mg/kg of organic Selenium (Org-Se) (2 g of crushed yeast; Sel-Plex 2000 mg Se kg-1, Alltech, Lexington, KY) according to^33^, and the BD with 0.3 mg/kg of nano-selenium (Nano-Se) according to^34^. The broodstock were fed these diets in triplicate over an 84-day period, from October 12 to January 4. Table 1 displays the components and chemical analysis of the diet used to feed G. Sea bream (*Sparus aurata*) broodstock during maturation.

### Blood samples

Samples were collected from the caudal veins of anesthetized broodstock. This study used live Gilthead Seabream broodstock, avoiding the need to euthanize the fish during the study period. 50 ppm of MS222 anesthetized the fish before injection and/or blood sampling. Samples were taken from both males and females in each replicate. At 4,000 rpm for 10 min at 4 °C, blood samples were centrifuged to separate the serum, which was then stored at -80 °C until hormonal and biochemical assays were performed.

### Spawning protocol

Samples of eggs were collected using a polyethylene cannula with an external diameter of 1.25 mm and an internal diameter of 0.86 mm to assess egg development based on egg diameter. The average egg diameter measured 580 microns. A hormonal injection of LHRHa (des-Gly10, [D-Ala6] LH-RH Ethyl amide, Sigma-Aldrich, St. Louis, Missouri, USA) was administered at ten micrograms per kilogram of fish body weight. The broodstock were injected intramuscularly with the hormone.

After 60–72 h of hormonal injection, the broodstock released eggs. Each tank was supported by a stand-up collector pipe for surface-drain eggs, equipped with a 400-micron screen. The collected eggs were recorded daily in an Excel sheet for each replicate, including the number of eggs, percentage of rotten eggs, good eggs, and fertilized eggs, along with other relevant information.

### Egg samples

After spawning, samples of eggs were taken for microscopic examination. A sample of the eggs collected from the egg collector can be examined microscopically to determine the diameter of the eggs and the diameter of the oil droplets.

### Larval rearing stage for 20 DPH

Eggs from each replicate were transferred to a 20-liter bottle for fifteen minutes to separate fertile from unfertile eggs. Ten thousand eggs per liter were stocked. One hundred thousand fertilized eggs from each replicate were then disinfected with Betadine before being transferred to nine larvae-rearing tanks, with 5 m³ in an active volume of 4 m³, for a 20DPH larvae-rearing experiment. Seawater, after filtration and sterilization, was used to fill the tanks, maintaining the exact temperature and salinity conditions.

### Preparing larval rearing tanks

In preparation for receiving the larvae, the greenhouse was maintained, washed, cleansed, and disinfected. The greenhouse contained nine 5 m^3^ fiberglass tanks covered in black polyethylene to provide adequate darkness for controlling the greenhouse lighting. The experiment included three treatments, each with three replications.

Newly hatched larvae were placed in separate tanks, each with a 4 m³ water volume. The stocking density was set at 25 larvae per liter in each larval rearing tank, with a daily water exchange rate of 25%. Throughout this period, the tanks were kept in complete darkness 2DPH. Green algae (*Nanochloropsis sp.*) were introduced at a density of 500,000 mL starting at 3 days post-hatching (3DPH) to enhance water quality. According to the feeding protocol, live feed (Rotifer and Artemia) was provided during the 20DPH of the larval rearing process. The schedule for feeding the larvae with live food and MD, as adopted from^35^, is shown in Table 2.

### Evaluation of larval rearing stage for 20 DPH

At 12 days post-hatching (DPH), 20 larvae from each replicate were sampled five times per tank for examination of swim bladder percentage under a microscope. Larvae were collected and counted to calculate the fish survival rate and assess larval growth in terms of weight and length at the end of the larval experiment, 20 days post-hatching (DPH). A sample of larvae was also used to measure GPx enzyme activity (U/mg protein).

### Measured parameters

#### Reproductive performance and hatching quality

*Relative* Fecundity (1000 eggs per kg BW) = # spawned eggs/ Mother’s weight (kg).

Fertilization (%) = 100*(Fertilized eggs / eggs # per sample).

Hatching (%) = 100*(hatched egg / Total fertilized eggs # in each treatment.

### Reproductive hormones

FSH; mIU/mL levels were measured in the serum of both males and females using a dependable quantitative sandwich enzyme-linked immunosorbent assay (ELISA) kit (Mybioscience, San Diego, CA, USA) that has a sensitivity of 0.1 mIU mL-1, as described in^36^. The luteinizing hormone (LH) levels in fish (in mIU/mL) were assessed using a dependable quantitative competitive ELISA Kit from Mybioscience in San Diego, CA, USA, which has a sensitivity of 1.2 mIU/mL^37^.

Testosterone levels (expressed in ng/mL) in male serum and estradiol (E2; expressed in pg/mL) in females were evaluated using a simple solid-phase enzyme immunoassay (ELISA) provided by DRG Diagnostics GmbH, Germany. This technique is founded on competitive binding, exhibiting sensitivities of 0.083 ng/mL for testosterone and 9.71 pg/mL for estradiol, respectively^38^.

### Biochemical parameters

The total concentration of protein (in g/dL) in female serum was assessed using the biuret method as detailed by^39^. Albumin concentrations (in g/dL) were measured using the bromocresol green technique outlined by^40^. The globulin level (in g/dL) was subsequently calculated by deducting the albumin concentration from the total protein concentration.

Cholesterol levels (mg/dL) were measured using enzymatic hydrolysis and oxidation, as described by^41^. The total lipid content (mg/dL) was measured through a reaction with sulfuric and phosphoric acids, combined with vanillin, resulting in a pink color^42^. Triglyceride levels (mg/dL) were measured calorimetrically using a quadruple enzymatic process^43^.

### Lysozyme determination

The agarose gel cell lysis assay was used to measure lysozyme-specific activity (µg mg⁻¹ protein) in the serum of both male and female subjects^44^. The method involved preparing lysoplates by dissolving 0.01% agarose in 0.0067 M PBS (pH 6.3) at 100 °C. After the agarose cooled to between 60 and 70 °C, 500 mg of a uniform suspension of Micrococcus lysodeikticus in 5 mL of saline was added to 1 L of the agarose and mixed well. Then, the agarose mixture was poured into plates. Next, 25 µL of each serum sample and standard lysozyme solutions were placed into the wells of the prepared plates. The plates were incubated at 28-30 ºC for 15 hours, and the sizes of the clear zones formed were measured afterward. These measurements were then graphed against the standards to determine the lysozyme concentration in the samples.

#### Statistical analysis

Mean ± SE values are used to present the experimental data. The collected data included reproductive hormones, serum biochemical analyses, reproductive performance indices, and parameters such as survival, growth, and glutathione peroxidase (GPx) in Gilthead Seabream larvae. One-way analysis of variance (ANOVA) was used to identify differences among treatments, followed by Duncan’s multiple-comparison test for the means, *P* < 0.05 indicated statistical significance. Statistical analyses were performed using Standard Version 22, SPSS Inc., Chicago, Illinois, for Windows.

## Results

### Water quality

Water quality parameters are monitored weekly. Water temperature is 19 ± 1.0 °C, salinity is 35 ± 0.5 ppt, pH is 7.7 ± 0.3, dissolved oxygen (DO) saturation is not less than 80% (more than 5.3 ppm), and NH_3_ is below 0.007 mg/L. There are no significant differences between replicates (*P* > 0.05). The water quality criteria are suitable for the broodstock of the marine fish.

#### Reproductive hormones

Table 3 presents the levels of reproductive hormones LHRH, FSH, testosterone (T), and estradiol (E) in male and female G. Sea Bream broodstock fed three diets: Control (C), Nano-Se, and Org-Se during the maturation period. The Nano-Se supplemented diet significantly increased these hormone levels in both males and females compared to the other treatments. Males fed the Nano-Se diet had the highest levels of LH (0.935 mIU/ml), FSH (1.275 mIU/ml), and testosterone (2.7 pg/ml). Additionally, the control group, which did not receive selenium supplementation, showed decreased values: LH at 0.535 mIU/mL, FSH at 0.69 mIU/mL, and testosterone at 1.15 pg/mL. These results represent increases of 74.8% for LH, 84.8% for FSH, and 134.8% for testosterone compared to the control group. Meanwhile, males fed the Org-Se diet had hormone levels of 0.61 mIU/mL for LH, 0.98 mIU/mL for FSH, and 1.45 pg/mL for testosterone. These values are significantly higher than those of the control group by 14.01%, 42.02%, and 26.08%, respectively.

Similarly, female reproductive hormones showed a consistent trend, with the Nano-Se group having LH levels of 2.245 mIU/mL, FSH levels of 3.1 mIU/mL, and estradiol levels of 66.5 pg/mL. These increases represent rises of 58.1%, 121.4%, and 98.5% for LH, FSH, and estradiol, respectively, compared to the control diet. In contrast, the Org-Se diet resulted in hormone levels of 1.825 mIU/mL for LH, 1.935 mIU/mL for FSH, and 52.00 pg/mL for estradiol in females. The values for brooders fed Org-Se were also significantly higher than those fed the control diet, with increases of 28.5%, 38.2%, and 55.2%, respectively.

A comparison of the results from the main treatments—nano Selenium (Nano-Se) and organic Selenium (Org-Se)—shows significant differences in hormone levels. In males fed the Nano-Se diet, hormone levels were higher than in those with the Org-Se diet, with increases of 53.3% for LH, 30.1% for FSH, and 86.2% for testosterone (T). In females, hormone levels also increased, with rises of 23.0% for LH, 60.2% for FSH, and 27.9% for estradiol (E). Additionally, including Nano-Se in the male seabream diet boosted LH by 27.08%, FSH by 43% in females, and estradiol by 32.1% in males by the end of the experiment compared to their initial levels.

#### Immunity and lipid profile analyses

Table 4 presents the serum biochemical analyses of Gilthead seabream *Sparus aurata* broodstock fed three diets, including different forms of Selenium, over an 84-day maturation period. Adding Selenium to the diet improved immune function compared to the control group. Notably, using Selenium in its nanoform produced better results than when administered in its organic form. With Nano-Se supplementation, male broodstock showed total protein levels of 4.25 g/dL, albumin of 1.98 g/dL, and globulin of 2.37 g/dL. These values were significantly higher than those of the control, with increases of 24.7% in total protein, 24.2% in albumin, and 25.3% in globulin. In contrast, males fed the Org-Se diet had total protein levels of 3.95 g/dL, albumin of 1.64 g/dL, and globulin of 2.27 g/dL. Although these values also surpassed the control, they showed increases of 18.9% for total protein, 8.5% for albumin, and 22.0% for globulin.

In contrast, female broodstock fed the Nano-Se-supplemented diet exhibited total protein levels of 4.65 g/dL, albumin of 1.83 g/dL, and globulin of 2.83 g/dL. These values were significantly higher than the control, with increases of 15.0% for total protein, 10.9% for albumin, and 19.7% for globulin. The females fed the Org-Se diet had total protein levels of 4.15 g/dL, albumin of 2.15 g/dL, and globulin of 1.9 g/dL. These values were also higher than the control for total protein by 4.8% and for albumin by 24.1%, but globulin decreased by 16.2% compared to the control.

The serum lipid profiles of broodstock fed the three diets are shown in Table 4. It was observed that Nano-Se and Org-Se diets influenced lipid profiles. In males fed a Nano-Se diet, cholesterol levels were 137.8 mg/dL, triglycerides 171.4 mg/dL, and total lipids 806.0 mg/dL. Compared to the control, these values indicate a 38.0% decrease in cholesterol, a 61.2% decrease in triglycerides, and a 42.5% decrease in total lipids. Similarly, females fed the Nano-Se diet had cholesterol levels of 56.9 mg/dL, triglycerides of 84.0 mg/dL, and total lipids of 517.2 mg/dL, representing reductions of 77.4%, 76.8%, and 61.0%, respectively, compared to the control group.

The Org-Se diet follows the same pattern as the Nano-Se diet. Males had values of 206.37 mg/dL for cholesterol, 422.0 mg/dL for triglycerides, and 1294.1 mg/dL for total lipids. These values show decreases of 7.2% for cholesterol, 4.5% for triglycerides, and 7.7% for total lipids compared to the control group. Meanwhile, females had values of 139.7 mg/dL for cholesterol, 287.4 mg/dL for triglycerides, and 928.3 mg/dL for total lipids. These indicate reductions of 44.5% for cholesterol, 20.7% for triglycerides, and 30.1% for total lipids relative to control.

#### The G. Sea Bream broodstock serum lysozyme concentration

**Fig. 2:**
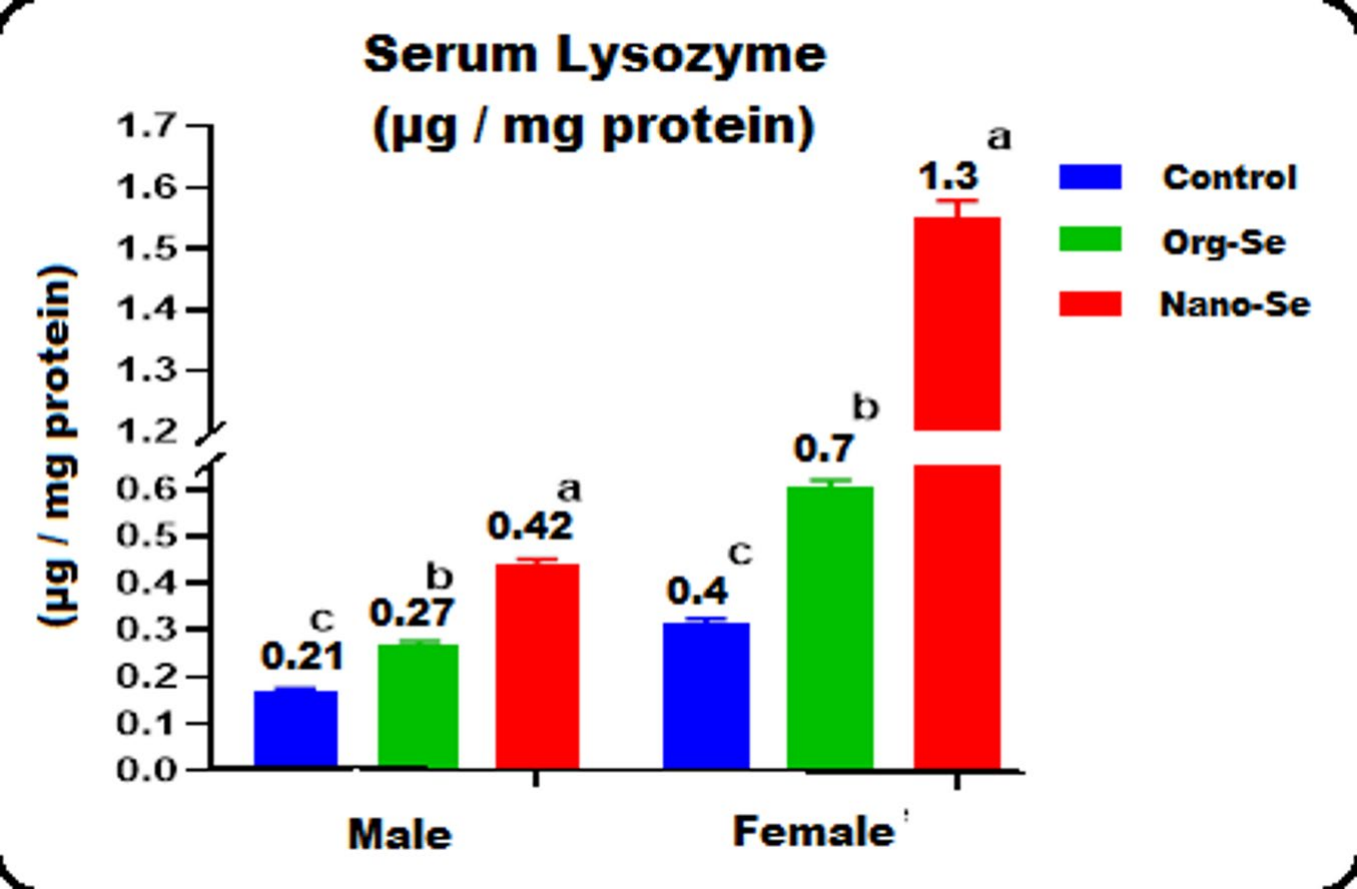
The concentration of lysozyme (µg / mg protein) in the serum of Gilthead seabream, *Sparus aurata* broodstock, fed three diets containing various forms of selenium during their maturation period.

Figure 2 illustrates the concentration of serum lysozyme of the Gilthead seabream, *Sparus aurata* broodstock fed the three treated diets during the maturation period. The Males fed Nano-Se and Org-Se diets showed significantly higher serum lysozyme levels, at 0.42 µg/mg protein and 0.27 µg/mg protein, respectively. In contrast, the control group had a serum level of 0.21 µg/mg protein. Similarly, females on these diets exhibited serum levels of 1.3 µg per mg protein for the Nano-Se diet and 0.7 µg per mg protein for the Org-Se diet, compared to 0.4 µg per mg protein for the control diet.

The improvements in males were notable, with increases of 158.8% for those fed with nano-selenium and 58.8% for those given organic-selenium. In females, the increases were even more remarkable, with improvements of 392.1% for nano-selenium and 90.6% for organic-selenium.

#### Reproductive performance

Table 5 shows the reproductive performance of G. Sea bream broodstock fed with three diets: Nano-Se, Org-Se, and Control (C) during the maturation period. The results reveal that selenium supplementation significantly improved several reproductive parameters compared to the control diet. Specifically, notable improvements were observed in relative fecundity (measured in thousands per kilogram of breeding females), fertilization rates, and hatchability, all at *P* ≤ 0.01.

The Nano-Se and Org-Se diets for G. Sea bream broodstock, compared to the control, showed significantly higher relative fecundity values, egg (1000)/female, of 1,432.6 and 1,341.0, respectively; the control had a fecundity value of 1,254.5, as shown in Table 5. This reflects increases of 14.2% and 6.9% over the control group, respectively.

Additionally, the fertilization rates for the Nano-Se and Org-Se groups were 82.88% and 81.81%, respectively, both higher than the control rate of 80.45%. The hatchability rates also showed significant improvement, with values of 96.0% and 94.3% for the Nano and Org selenium groups, respectively, compared to the control’s hatchability of 93.0%, reflecting increases of 3.2% and 1.4%.

The final egg and oil droplet diameters were significantly larger with selenium supplementation than in the control (*P* < 0.05). The Nano-Se data for these measures were 801.3 μm (egg diameter) and 187.0 μm (oil droplet diameter), while the Org-Se data were 797.0 μm and 185.0 μm, respectively. The control group recorded values of 787.0 μm for egg diameter and 180.3 μm for the oil droplet diameter.

#### Larval rearing efficiency

Table 6 displays the survival, growth, and swim bladder percentage of G. seabream, *Sparus aurata* larvae weighing 0.2 mg IBW for 20 DPH, produced from broodstock fed control (C), Nano-Se, and Org-Se diets during the maturation period. It was observed that Selenium supplementation in the mothers’ diets significantly enhanced both the growth parameters and survival rates of the larvae (*P* ≤ 0.01), and the swim bladder formation rate (*P* ≤ 0.05).

Specifically, the larvae showed significantly greater final lengths of 5.0 mm and 4.63 mm, along with length gains of 2.1 mm and 1.73 mm, for those produced from broodstock fed the Nano-Se and Org-Se diets, respectively, compared to larvae from broodstock fed the control diet (*P* < 0.05). Additionally, larvae from broodstock fed the Nano-Se diet had a final weight of 2.84 mg, a weight gain of 2.64 mg, a specific growth rate (SGR) of 13.27%/d, and a survival rate of 57.41%, surpassing the control group, which had measurements of 2.22 mg, 2.02 mg, 12.04%/d, and 53.8% for the same parameters. The Org-Se diet resulted in 2.54 mg, 2.34 mg, 12.71% per day, and 54.79%, respectively. The data for the Org-Se diet did not differ significantly from those of the Nano-Se diet or the control. Furthermore, larvae from broodstock fed the Nano-Se diet showed better outcomes than those from the Org-Se diet. The increases in data for the Nano-Se diet relative to the control diet were 22.0%, 75.0%, 27.9%, 30.7%, 10.2%, and 6.7%, respectively.

The swim bladder formation rates for larvae from broodfish fed Nano-Se and Org-Se diets are 98.0% and 97%, respectively. These results are significantly higher than those of brooders fed the control diet, which has a rate of 94.3% (*P* < 0.05).

#### Glutathione peroxidase (GPx) concentration in G. Sea Bream larvae

**Fig. 3:**
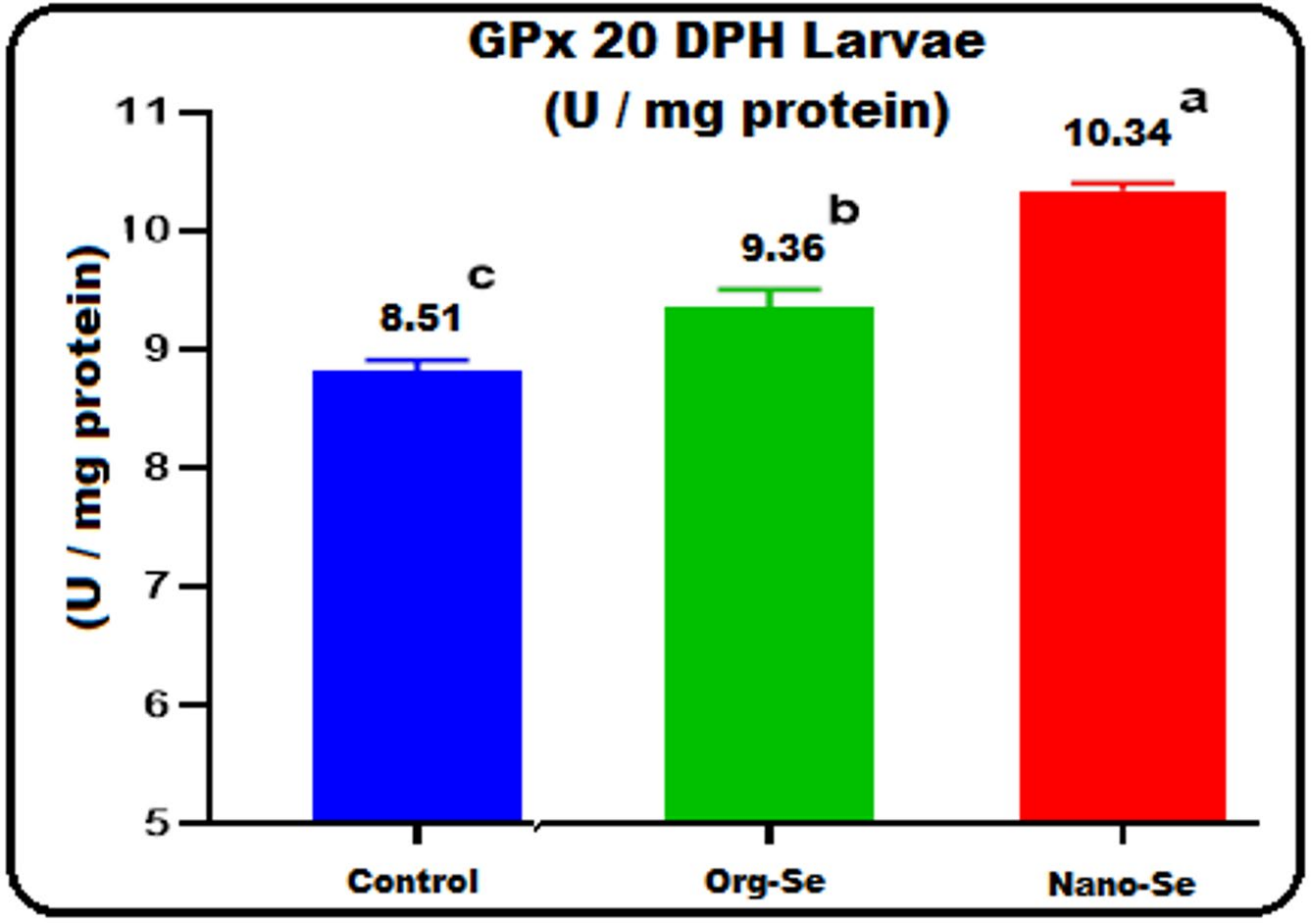
The concentration of glutathione peroxidase (GPx) (U/mg protein) in Gilthead seabream (*Sparus aurata*) larvae (20-DPH) produced from broodstocks fed the three diets containing various forms of selenium during the maturation period.

Figure 3 shows the impact of enriching G. Sea bream broodstock diets with Nano-Se and Org-Se on the levels of the key antioxidant GPx in their larvae 20 days after hatching. The diet with Nano-Se led to a significantly higher GPx level of 10.34 U/mg of protein (*P* ≤ 0.05) compared to the Org-Se diet (9.36 U/mg of protein), and the control group (8.81 U/mg of protein). Additionally, incorporating Selenium in its nanoform raised GPx levels by 17.4% relative to the control. Likewise, larvae from broodstock fed the organic Selenium diet showed a 6.2% increase in GPx compared to the control group.

## Discussion

The present work found increased levels of the hormones LHRH, FSH, testosterone (T), and estradiol (E2) in both male and female fish fed Nano-Se and Org-Se diets compared with those fed the control diet. At the same time, the Nano-Se diet was superior to the Org-Se diet in these criteria. This finding aligns with research by^45^, which demonstrated that female red tilapia fed a diet supplemented with NP-Se exhibited significantly higher weights for their hepatic, visceral, and gonadal tissues than those in the other groups. They also found that fish consuming the NP-Se diet exhibited elevated levels of luteinizing hormone, progesterone, and estradiol, while showing the lowest levels of follicle-stimulating hormone. However, they were followed by the fish that consumed the Na_2_SeO_3_ + NP-Se and Na_2_SeO_3_ diets. The secretion of reproductive hormones and the regulation of the reproductive cycle in fish are still poorly understood. The study by^46^ explored the potential role of gonadotropin secretion in European sea bass. They discovered that regulating gonadotropin secretion involves complex processes, including mechanisms by which the brain steroids, mainly through the GnRH system, provides feedback.

The importance of the nutritional effect on the reproductive processes of fish was emphasized by^47^. In this situation, the survival, development, and reproductive capacity of larvae are significantly impacted by nutrition, as observed by^48,50,50^. Like other vertebrates, reproductive hormones, such as LH and FSH, play essential roles in promoting gonadal development in teleost fish^51^. For instance, FSH is predominant during spermatogenesis. Simultaneously, during spermatogenesis in males and salmon (*Salmo salar*) broodstock, oocyte maturation and ovulation, in females, the levels of LH increase^52^. Additionally, research by^53^ observed that reproductive hormones of Nile tilapia broodstock increased in fish fed a diet supplemented with 1 mg nano Selenium (NSe) per kilogram along with vitamin E (VE) at 100 mg of the basal diet. Moreover, plasma FSH levels in male brooders of the sea urchin are associated with testosterone and androgen levels; in both genders, the circulating LH levels correlate with the reproductive hormone levels of females^54^. Moreover, findings by^55^reported increased gonadotropic hormone levels in red tilapia brooders, supporting the idea that NP-Se may enhance the pituitary gland’s receptor expression of the GnRHR hormone. They also explain the physiological processes of Selenium regarding various energy balance, through its diverse functions in redox reactions. Selenium is crucial for the production of selenoproteins, which play antioxidant roles. Variations in Selenium levels have been linked to disruptions in homeostasis and the functions of selenoproteins, indicating a significant effect on energy regulation. Additionally, Selenium is vital for the production of sex hormones like testosterone, estradiol, and progesterone^56^.

Adding the two forms of Selenium (Nano-Se and Org-Se) to the basal diet in the present study, as compared to the control, significantly increased albumin, total protein, and globulin levels in both males and females (*P* < 0.01). Our findings are consistent with the work of^53^. In their study, a dietary combination of 1 mg of NSe per kilogram and 100 mg of vitamin E per kilogram (G3) was examined in Nile tilapia broodstock. Results showed that blood parameters (RBC, WBC, PCV, and hemoglobin) were significantly elevated (*P* < 0.05). They also revealed that, as compared with the control (G0), albumin, total protein, and globulin levels increased. These diets consisted of a basal diet and individual treatments of 1 mg NSe/kg (G1) and 100 mg VE/kg (G2). Studies on *Paramisgurnus dabryanus* conducted by^57^ and on *Clarias gariepinus*, by^11^, they found that adding NSe to the diet resulted in decreased levels of Alanine aminotransferase and aspartate aminotransferase. Research has also been conducted by^58^ on common carp (*Cyprinus carpio*), and has arrived at similar findings. They observed an improvement in the activities of ALT, AST, and ALP, as well as total protein levels, with dietary supplementation of 3 mg NSe/kg. An increase was observed by^59^ in RBCs, Hb, PCV, and WBCs of European seabass given various concentrations of NSe, as well as total protein, albumin, and globulin levels in the serum, compared to the untreated group.

The serum lysozyme concentration in both male and female G. Sea bream broodstock fed the Nanoparticle and Organic selenium compared to the control diet in the present study increased significantly during the maturation period. Similarly, albumin, total protein, and globulin levels also rose considerably in response to the same treatments. Our results align with those of^60^. They found that serum lysozyme and hemolytic activities, as well as the activity of white blood cells involved in the respiratory burst, were higher in fish fed Se-NP-supplemented diets compared to the control. They also found that the globulin, total protein, and the ratio between globulin and albumin in the serum were higher in fish-fed SeNP1, 2, and 4 diets as compared to the other groups (*P* < 0:05). The presence of Selenium in the diets of certain fish is essential for the growth and immunity^61^. They stated that the biological functions of fish are directly affected by the dietary vital role of selenium, including the increase in respiratory burst, as well as the enhancement of lysozyme, acetylcholinesterase, and myeloperoxidase activity. It was found that the expression of fatty acid oxidation genes and liver triglyceride hydrolysis increased as the diets were supplemented with Selenium^62^.

The present study indicates that cholesterol, triglycerides, and total lipids were significantly reduced in both males and females fed selenium-fortified diets, using both nano and organic forms of selenium (*P* < 0.01). A study by^63^found that feeding male rats a diet supplemented with one part per million (ppm) Selenium, along with 2% cholesterol, significantly decreased the apolipoprotein B (apo B) levels and the 3-hydroxy-3-methylglutaryl coenzyme A (HMG-CoA) reductase expression after three months of treatment. In a similar study^64^, found that adding VE and Selenium nanoparticles to the diets of rainbow trout, either separately or in combination, compared to the control, resulted in reduced cholesterol levels. Additionally, it was found that including VE and Selenium in the diet of largemouth bass could safeguard tissues from oxidative damage resulting from the oxidation of dietary oils, as shown in the findings of^65^. Research by^66^indicated that supplementing the diet with selenium resulted in lower lipid levels in both the liver and muscles when fed an oil oxidation-enhanced diet. Moreover^65^, noted that largemouth bass consuming diets with fresh oil showed increased liver lipid accumulation when supplemented with C 1.42 mg kg^- 1^ of selenium. It appears that the provision of selenium in largemouth bass may enhance the metabolic processes related to lipogenesis, and further exploration requires an explicit understanding of the underlying mechanisms. In general, considerable attention has been given to protecting embryonic and larval stages, along with the addition of Se to the maternal diet^67^.

The present study shows that a diet enriched with nano- or Organic selenium significantly increased the total egg production per female, relative fecundity, fertilization, and hatchability (*P* < 0.01). The diameters of the egg and oil droplets, as compared to the control diet, increased significantly with the Nano-Se and Org-Se diets (*P* < 0.05). The reproductive performance of broodstock is affected by their nutrition^68,69^. It was found that female Arabian yellowfin sea bream (*Acanthopagrus arabicus*) exhibited the highest fertilization rate (FR) when they were fed a plant protein-rich diet fortified with 4 mg of nanoparticle selenium per kg diet, compared to the control group and the group fed the diet fortified with 2 mg of N-Se per kg^70^. This combination is also essential for optimal reproductive capacity. In groups supplemented with NSe, reproductive performance improved, as evidenced by an increase in the number of fries produced per female and the weight of the fry. The fish that were fed the NSe/VE blend demonstrated the best results in terms of gonadal architecture. Additionally, the results of^71^ found that feeding females (*O. niloticus*) a diet with VE, zinc, and selenium exhibited higher relative fecundity in comparison to a control diet for 144 days during the spawning season. They also recorded high values as compared to the control of relative fecundity with the VE + Zn and VE + Se treatments.

In conclusion, our findings indicate that incorporating Nano-Se at 0.3 mg/kg or Org-Se at 4 mg/kg into the basal diets can enhance the reproductive outcomes of female G. Sea bream. This supplementation increases the total number of eggs, egg size, fertilization success, and hatchability rates. These results align with earlier research, which has shown that feeding *A. arabicus* broodfish plant-based diets supplemented with Nano-Se improves larval quality by reducing embryo defects and increasing the size and survival rates of both hatchlings and larvae^70^. Moreover, it has been shown that maternal selenium (Se) intake affects the overall selenium levels in both eggs and larvae. A higher hatchability rate was also seen in the post-larvae of *A. arabicus*. Additionally, feeding Convict cichlid fish (*Amatitlania nigrofasciata*) a diet containing VE at 100 mg per kilogram and Se nanoparticles at 0.1 mg per kilogram^72^. found that female reproductive metrics, such as maximum ovum size, egg diameter, and hatching success, were enhanced. According to^73^ the optimal Se level in female fish brooders has a positive impact on egg survival and hatching rate. These authors suggested that incorporating dietary Selenium nanoparticles may enhance reproductive activities and improve the physiological condition of fish, resulting in better broodstock spawning performance.

The present study demonstrates that incorporating selenium into a mother’s diet, in both nano and organic forms (Nano-Se and Org-Se), significantly enhanced the growth parameters and survival rates of the larvae of G. Sea bream (*P* ≤ 0.01), as well as the formation rate of swim bladders (*P* ≤ 0.05). These findings align with the results of^74^. They reported that a High Se diet resulted in higher mean weight values and condition factors of *Cyprinus carpio* compared to a Low Se diet after 60 days. Also, with the Nano-Se diet, the number of spawning females increases. Reproductive performance and physiological responses improve, along with higher fecundity, fertility, egg diameter, reproductive hormones, the quality of eggs and sperm, survival rates, and a greater number of fries^70,75^. It was also observed that supplementing diets with NSe improved the reproductive performance of rainbow trout broodstock by increasing fertilization, the quality of eggs and sperm, and offspring survival^76^.

The present work results illustrate that fortifying G. Sea bream broodstock diets with Nano and Organic Selenium significantly affects the critical antioxidant GPx enzyme activity in their 20-day post-hatch larval bodies. The diet that included Nano-Se and Org-Se resulted in a considerably higher glutathione peroxidase (GPx) concentration in 20 DPH larval bodies compared to the control. Our results align with those of^77,79,79^. The biosynthesis of selenocysteine needs Selenium as an essential element for the activation of GPx enzymes^80,82,82^. The GPx enzyme is critical for protecting cells against lipid peroxidation, as shown by^3^. Selenium plays a role in eliminating hydrogen peroxide (H_2_O_2_) and lipid peroxides found in the cytosol of cells and the mitochondrial matrix. These biological actions contribute to the preservation of membrane integrity and protect biomolecules, such as lipids, lipoproteins, and nucleic acids, from oxidative harm induced by both biological and environmental stressors^83^. Selenium is integrated into protein synthesis as a selenocysteine residue, leading to the formation of antioxidant enzymes, such as glutathione peroxidase (GPx)^84,85^. Key selenium-dependent enzymes, including glutathione peroxidase (GPX), thioredoxin reductase (TR), and methionine sulfoxide reductase (MSR), are recognized for their antioxidant properties. They protect against oxidative damage by neutralizing free radicals and reducing the harm inflicted on lipids and other biological materials^86^.

Our findings are consistent with those of^87^, who discovered that using Selenium nanoparticles in fish under various stress factors can enhance growth performance and improve antioxidant status. Previous studies have confirmed that dietary nano-selenium (NSe) is more effective than its organic and inorganic counterparts. This effectiveness is attributed to the higher rate of absorption, low toxicity, higher bioavailability, and higher activity of biological functions in the fish digestive system^58,87^. In a previous study involving broodstock, researchers found that plant protein-based diets supplemented with Selenium did not impact the broodstock glutathione peroxidase (GPX) activity. However, it increased the swim-up larval GPX activity levels^88^. Additionally, selenium-supplemented diets for rainbow trout fry have a positive effect on the activity of GPX^23^.

## Conclusion

Based on the results of the presented study, diets fortified with 0.3 mg/kg of Nano-Se or 4 mg/kg of Org-Se in *Sparus aurata* broodstock, fed for 84 days, showed improvements in reproductive performance, biochemical analyses, immune parameters, larval survival, larval growth, and antioxidant capacity in the offspring. The study could be a significant topic in aquaculture nutrition with potential applications for broodstock management.

**Table 1:** Components and chemical analysis of the diets used to feed G. sea Bream (*Sparus aurata*) broodstock during the maturation period.

Feed ingredients %	Control	*N*-Se	O-Se
Wheat flour	10.00	10.00	10.00
Wheat bran	2.40	2.40	2.40
Maize gluten	10.00	10.00	10.00
Soybean meal	20.00	20.00	20.00
Yellow corn	5.00	5.00	5.00
Fish meal	40.00	40.00	40.00
Fish oil	8.00	8.00	8.00
CMC^a^	3.00	3.00	3.00
Selino amino acid	0.20	0.20	0.20
Vit. & min mix.^b^	1.00	1.00	1.00
Vit. C	0.40	0.40	0.40
TOTAL	100.00	100.00	100.00
Proximate analyses			
Crude protein	45	45	45
Lipids	15	15	15
Fiber	1.5	1.5	1.5
Ash	8	8	8
NFE^c^	23.5	23.5	23.5
Total energy/100 g	491.98	491.98	491.98
Selenium (mg/100 g)	0.106	0.106	0.106

^a^CMC is a carboxymethylcellulose.

^b^Vitamin and mineral mixture per Kg Premix: Vitamin A = 4.8 × 106 IU; D3 = 0.8 × 106 IU; Vitamin E = 4 g; Vitamin K = 0.8 g; Vitamin B1 = 0.4 g; Riboflavin = 1.6 g; Vitamin B6 = 0.6g; Vitamin B12 = 4 mg; Pantothenic acid = 4 g; Nicotinic acid = 8 g; Folic acid = 0.4g; Biotin = 20 mg; Choline chloride = 200 g; Cu = 4 g; I = 0.4 g; Iron = 12 g; Mn = 22 g; Zn = 22 g; Selenium = 0.4 g.

^c^NFE is a nitrogen-free extract.

**Table 2:** Feeding schedule for larval rearing from 2 DPH to 20 DPH for sea Bream showing microalgae (Cell /ml), rotifers, and artemia (individual/ml), and dry feed applied throughout the day (adopted from Moretti et al., 1999).

Period DPH		2–17	18–20	20 to the end of the weaning period 55 DPH		
Micro algae (cell /ml)		500		Increased to 800,000	800,000 in 33 DPH, then decreased	
Sea bream	Rotifer	5 / ml Gradually increased to 15				
	*Artemia*	0	0.2 increased to 1.6		1.6 in 40 DPH	
	Dry feed			Starter 100–200 µ after 20 DPH		

**Table 3:** The reproductive hormones of Gilthead seabream, *Sparus aurata*, bloodstock fed different forms of selenium during maturation.

Measured parameters		Initial sample*	Final sample**			*P-*Value	
			C	Nano-Se	Org-Se		
Male	LHRH (mIU ml^-1^)	0.480 ± 0.006	0.535 ± 0.014^c^	0.935 ± 0.009^a^	0.61 ± 0.006^b^	0.000	
	FSH (mIU ml^-1^)	0.505 ± 0.003	0.69 ± 0.012^c^	1.275 ± 0.009^a^	0.98 ± 0.006^b^	0.000	
	Testosterone (pg ml^-1^)	0.925 ± 0.009	1.15 ± 0.029^c^	2.7 ± 0.058^a^	1.45 ± 0.029^b^	0.000	
Female	LHRH (mIU - 1 ml)	0.960 ± 0.006	1.42 ± 0.006^c^	2.245 ± 0.009^a^	1.825 ± 0.014^b^	0.000	
	FSH (mIU ml - 1)	1.25 ± 0.029	1.4 ± 0.006^c^	3.1 ± 40.058^a^	1.935 ± 0.009^b^	0.000	
	Estradiol (pg ml - 1)	24.5 ± 0.289	33.5 ± 0.289^c^	66.5 ± 0.289^a^	52 ± 1.155^b^	0.000	

*Initial sample before experimental start.**The final sample is taken one hour before injecting the spawning hormone. Values in the same row with a different superscript are significantly different (P ≤ 0.01).

**Table 4:** Serum biochemical analyses of Gilthead seabream *Sparus aurata* broodstocks fed different forms of selenium during the maturation period.

Measured parameters	Male				Female				
	Control	Nano-Se	Org-Se	*P*-value	Control		Nano-Se	Org-Se	*P*-value
Total Protein (g dl^-1^)	3.20 ± 0.058^c^	4.25 ± 0.029^a^	3.95 ± 0.029^b^	0.001	3.950 ± 0.03^c^		4.65 ± 0.03^a^	4.150 ± 0.03^b^	0.01
Albumin (g dl^-1^)	1.50 ± 0.012 ^c^	1.98 ± 0.014^a^	1.64 ± 0.014^b^	0.01	1.635 ± 0.01^c^		1.83 ± 0.01^b^	2.150 ± 0.03^a^	0.001
Globulin (g dl^-1^)	1.77 ± 0.009^c^	2.37 ± 0.009^a^	2.27 ± 0.017^b^	0.01	2.270 ± 0.02^b^		2.83 ± 0.01^a^	1.900 ± 0.06^c^	0.001
Cholesterol (mg dl^-1^)	222.5 ± 1.44^a^	137.80 ± 1.44^c^	206.37 ± 1.44^b^	0.01	252.0 ± 1.15^a^		56.90 ± 1.44^c^	139.70 ± 1.44^b^	0.01
Triglycerides (mg dl^-1^)	442.0 ± 5.77^a^	171.4 ± 3.02^b^	422.0 ± 2.89^a^	0.001	362.83 ± 4.34^a^		84.0 ± 3.71^c^	287.43 ± 2.84^b^	0.001
Lipid (mg dl^-1^)	1402.0 ± 1.7 ^a^	806.0 ± 2.3^c^	1294.1 ± 2.0^b^	0.01	1329.0 ± 1.7^a^		517.2 ± 1.2^c^	928.3 ± 2.0^b^	0.001

Values in the same row with a different superscript are significantly different (*P* ≤ 0.05).

**Table 5:** Reproductive performance (fecundity and hatching) of Gilthead seabream, *Sparus aurata* broodstock fed with different forms of selenium during the maturation period.

Treatment	Control	Nano-Se	Org-Se	*P*-value
Average female weight, kg	1030.33	1010.50	1034.33	NS
Total Number of eggs (1000)/female	1292.5 ± 12.99^c^	1447.67 ± 13.29^a^	1387.0 ± 10.39^b^	0.000
Relative fecundity, # of eggs (1000)/kg female	1254.5 ± 2.10^c^	1432.6 ± 5.88^a^	1341.0 ± 1.60^b^	0.000
Fertilization (%)	80.45 ± 0.2^c^	82.88 ± 0.3^a^	81.81 ± 0.1^b^	0.01
Hatchability, %	93.0 ± 0.58^c^	96.0 ± 0.58^a^	94.3 ± 0.33^b^	0.01
Final egg diameter (µm) after spawning	787 ± 1.15 ^b^	801.3 ± 2.19 ^a^	797 ± 1.2 ^a^	0.050
Oil droplet diameter (µm)	180.3 ± 0.67^b^	187 ± 1.15^a^	185 ± 0.58^a^	0.050

Values in the same row with a different superscript are significantly different.

**Table 6:** Survival, growth, and swim bladder % of G. seabream, *Sparus aurata* larvae 0.2 mg IBW for 20 DPH produced from broodstock fed at control (C), Nano-Se, and Org-Se diets during the maturation period.

Treatment	Control	Nano-Se	Org-Se	*P*-Value
Final length, mm*	4.10 ± 0.06^c^	5.00 ± 0.12^a^	4.63 ± 0.07^b^	0.001
Length gain, mm	1.20 ± 0.06^c^	2.10 ± 0.12^a^	1.73 ± 0.07^b^	0.001
Final weight, mg	2.22 ± 0.06^b^	2.84 ± 0.001^a^	2.54 ± 0.01^ab^	0.001
Gain, mg	2.02 ± 0.06^b^	2.64 ± 0.003^a^	2.34 ± 0.01^ab^	0.001
SGR, %/day	12.04 ± 0.14^b^	13.27 ± 0.01^a^	12.71 ± 0.02^ab^	0.001
Survival, %	53.80 ± 0.45^c^	57.41 ± 0.63^a^	54.79 ± 0.26^b^	0.004
Swim bladder percentage, %	94.33 ± 0.33^b^	98 ± 0.58^a^	97 ± 0.58^a^	0.050

*Initial length = 2.9 mm/larvae. Values in the same row with a different superscript are significantly different.

## Data Availability

The datasets used and/or analyzed during the current study are available from the corresponding author on reasonable request.
